# The assembly and characterisation of two structurally distinct cattle MHC class I haplotypes point to the mechanisms driving diversity

**DOI:** 10.1007/s00251-015-0859-9

**Published:** 2015-07-31

**Authors:** John C. Schwartz, John A. Hammond

**Affiliations:** Livestock Viral Diseases Programme, The Pirbright Institute, Ash Road, Pirbright, Woking, Surrey, GU24 0NF UK

**Keywords:** MHC class I, Cattle, Bovine, Evolution, Antigen presentation

## Abstract

In cattle, there are six classical *MHC class I* genes that are variably present between different haplotypes. Almost all known haplotypes contain between one and three genes, with an allele of *Gene 2* present on the vast majority. However, very little is known about the sequence and therefore structure and evolutionary history of this genomic region. To address this, we have refined the *MHC class I* region in the Hereford cattle genome assembly and sequenced a complete A14 haplotype from a homozygous Holstein. Comparison of the two haplotypes revealed extensive variation within the *MHC class Ia* region, but not within the flanking regions, with each gene contained within a conserved 63- to 68-kb sequence block. This variable region appears to have undergone block gene duplication and likely deletion at regular breakpoints, suggestive of a site-specific mechanism. Phylogenetic analysis using complete gene sequences provided evidence of allelic diversification via gene conversion, with breakpoints between each of the extracellular domains that were associated with high guanine-cytosine (GC) content. Advancing our knowledge of cattle *MHC class I* evolution will help inform investigations of cattle genetic diversity and disease resistance.

## Brief communication

Major histocompatibility complex (MHC) class I genes encode transmembrane glycoproteins which present peptides from intracellular proteins on the cell-surface. These peptide/MHC complexes are recognised by receptors on CD8+ T-cells and natural killer (NK) cells, and thus play a central role in cell-mediated immune responses. Allelic diversity and variable gene content are important functional characteristics of MHC genes, believed to be primarily driven by selection from rapidly evolving pathogens (Doxiadis et al. 2011; Kelley et al. 2005; Parham et al. 1995). As a consequence, the MHC locus can differ substantially in gene content and organisation even between closely related species (Kelley et al. 2005). In cattle, there are six described polymorphic classical class I (*MHC class Ia*) genes (Hammond et al. 2012) encoded within the MHC on chromosome 23, all of which encode functional antigen-presenting transmembrane proteins (Gaddum et al. 2003; Graham et al. 2008; Guzman et al. 2010; Guzman et al. 2008; MacHugh et al. 2009). As well as allelic polymorphism, cattle *MHC class I* haplotypes also vary in gene content; only one to three of these genes are present on any given haplotype, with *Gene 2* being the most ubiquitous (Birch et al. 2006; Codner et al. 2012; Ellis et al. 1999).

The allelic diversity of Holstein cattle *MHC class I* has been well studied; however, very little is known about the full gene sequences and underlying genomic structure between the numerous different haplotypes. The cattle genome contains the most complete full haplotype sequence, assembled using DNA sequence from a Hereford cow (L1 Dominette 01449) and her sire (L1 Domino 99375) (Elsik et al. 2009). This extended *MHC class I* region was described by Birch et al. (2008) and reviewed by Ellis and Hammond (2014). Briefly, the genome contains two *MHC class Ia* genes, a *Gene 2* and a *Gene 5*. Centromeric are five pseudogenes and the non classical gene *NC1*. However, considering the heterozygous and repetitive nature of the MHC and that there may be three haplotypes in the assembled data, this region of the genome assembly is considered putative. This is particularly evident at the telomeric end, as both current assemblies of the same cattle genome data differ in gene position and content (Ellis and Hammond 2014). The only other haplotype to be structurally characterised is the Holstein A14 haplotype which was mapped using a bacterial artificial chromosome (BAC) library from a homozygous dairy bull (Di Palma et al. 2002). This haplotype contains three *MHC class Ia* genes (5′ to 3′: *Gene 2*, *Gene 4*, and *Gene 1*) and a possible fourth gene (historically referred to as Gene “*Z*”). To compare these divergent haplotypes and begin to assess how cattle *MHC class I* has evolved, we refined the Hereford *MHC class I* genome assembly and assembled the Holstein A14 haplotype using modern sequencing and bioinformatics methods.

To refine the Hereford genome *MHC class Ia* region, we exploited L1 Domino BAC clones that were previously sequenced as part of the cattle genome project (Elsik et al. 2009). Four fully sequenced and largely assembled clones which overlap the *MHC class Ia* locus were identified: CH240-252J17, CH240-271N5, CH240-103G5, and CH240-463M1 (GenBank: FQ482148, FO681480, FQ482089, and AC182900, respectively). Our analysis of these clones using the original raw Sanger sequencing reads confirmed their overall assembly but identified base pair calling errors at the end of the clones that created some false single nucleotide polymorphisms (SNPs) and indels. For clone CH240-463M1, we could not improve on the current *de novo* assembly of nine unordered contigs. Based on sequence identity to the three complete clones, we were able to order these nine contigs with high confidence. Gaps between each contig ranged from 1 to 4610 bp suggesting that relatively little sequence information was missing. Comparison between all four clones revealed an identical 12.6-kb overlap between CH240-252J17 and CH240-271N5, and an almost identical 72.3-kb overlap between CH240-463M1 and CH240-103G5, except for ten positions within 200 bp of the contig ends. The 46.5-kb overlapping region between CH240-463M1 and CH240-271N5/252J17 was also almost identical (including the *Gene 2* locus) except for a 514-bp inversion, two significant insertion/deletions (798 and 201 bp) and 56 SNPs. However, all of these differences were located within 2 kb of the clone ends and were almost all previously identified by our analysis of the raw sequence data as incorrectly annotated. Consequently, we are confident that these four clones represent a single *MHC class I* haplotype.

**Fig. 1:**
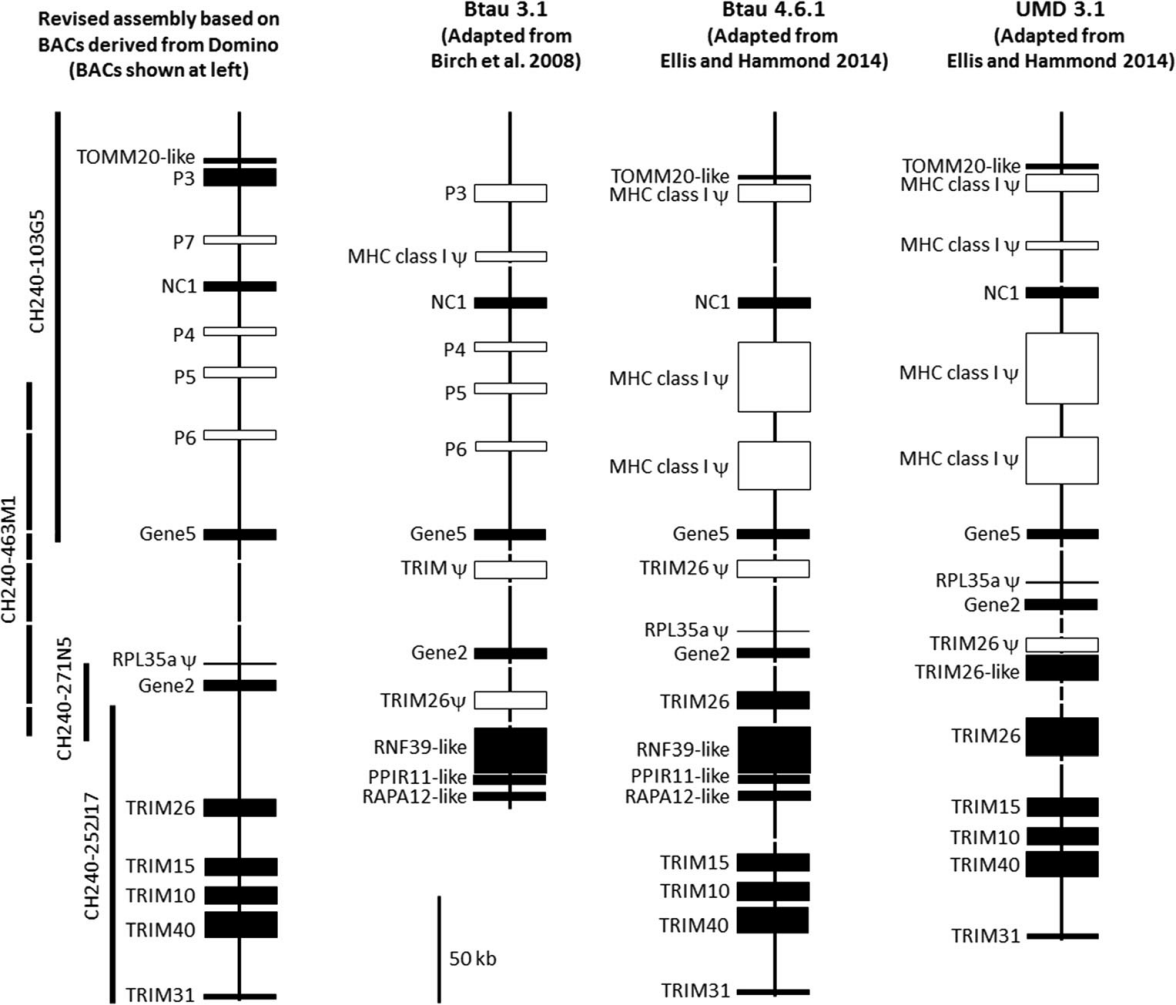
Comparison of previously published cattle genome assemblies (Birch et al. 2008; Ellis and Hammond 2014) with the assembly in this study. Gaps within each assembly are indicated by *broken lines. Closed boxes* represent putatively functional genes, *open boxes* (and *RPL35aψ*) represent pseudogenes. BACs used for the current analyses are shown at *left*

A comparison between the current genome builds (Btau 4.1 and UMD 3.1) and the assembled BAC clones reveals that the current assemblies, although broadly correct, likely contain some significant errors (Fig. 1). Although the order of the *MHC class I* genes and pseudogenes is mostly correct, the number and size of several pseudogene loci and intergenic intervals are substantially different, particularly between *NC1* and *Gene 5*. Of note is *pseudogene 3* which is a putatively functional gene in our assembly, although the large intron 1 and 2 sequences (2853 and 1971 bp, respectively) are likely to impact negatively on expression. We also found no evidence of *TRIM26* pseudogenes between *Gene 2* and *Gene 5*, and a less gene dense region telomeric of *Gene 2* that only contains functional TRIM genes. It is likely that the sequence data from more than one haplotype has been incorrectly incorporated into the genome assemblies creating assembly and annotation problems.

To sequence a complete Holstein A14 *MHC class Ia* haplotype, the same five BAC clones from the previous study (Di Palma et al. 2002) were isolated, expanded overnight, and BAC DNA was harvested using the Qiagen Large Construct Kit (Qiagen, GmbH, Hilden, Germany). Purified BAC DNA from four of the clones (108G12, 307D8, 251E3, and 370E1) was sequenced using an Illumina MiSeq with 250 × 250 bp paired-end reads prepared using the Nextera Mate Pair Sample Preparation Kit (gel-free method) (Source Biosciences, Inc. Nottingham, UK) and *de novo* assembled using Velvet (Zerbino and Birney 2008). The remaining clone (175E3) was sequenced using the Pacific Biosciences RSII platform (mean read length >8 kb) (GATC Biotech AG, Koblenz, Germany). Read filtering and assembly was conducted using the Pacific Biosciences SMRT Analysis software (v2.3.0; http://www.pacb.com/devnet/). Gene content was queried using BLAST (Altschul et al. 1990) against GenBank, all 114 known cattle *MHC class Ia* and *MHC class Ib* cDNA alleles contained within the Immuno Polymorphism Database (IPD-MHC; Release 1.3.0 (18 Nov. 2011)); http://www.ebi.ac.uk/ipd/mhc/bola; (Robinson et al. 2013), and all of the genes in the extended *MHC class I* region in the genome assemblies. The final haplotype was annotated using Artemis (Rutherford et al. 2000) and has been submitted to GenBank (accession number KT005248).

To confirm our haplotype assembly, we compared an *in silico*-derived HindIII restriction fragment profile for each assembled BAC clone, to the previous BAC clone mapping conducted by Southern blot hybridization of HindIII fragments (Di Palma 1999). All clones matched the previously observed profiles including a discrepancy between the overlapping clones 251E3 and 370E1. These clones differ in length between the two most 3′ HindIII fragments, suggesting possible heterozygosity at this end of the locus. These discordant BACs are identical over approximately 50 kb, but there are 163 SNPs and 16 indels over the 20.4-kb overlap at the 3′ end. Of these, 23 SNPs lie within the coding region of *NC1*, of which 17 are non-synonymous, with both sequences matching the previously known alleles NC1*00501 and NC1*00601. In contrast, comparison of 370E1 with our Hereford BAC assembly reveals only 15 SNPs over approximately 98 kb of overlap, none of which lie within the coding region of *NC1*. Thus, these animals from different breeds both carry NC1*00501, with the Holstein animal being heterozygous and also carrying NC1*00601.

**Fig. 2:**
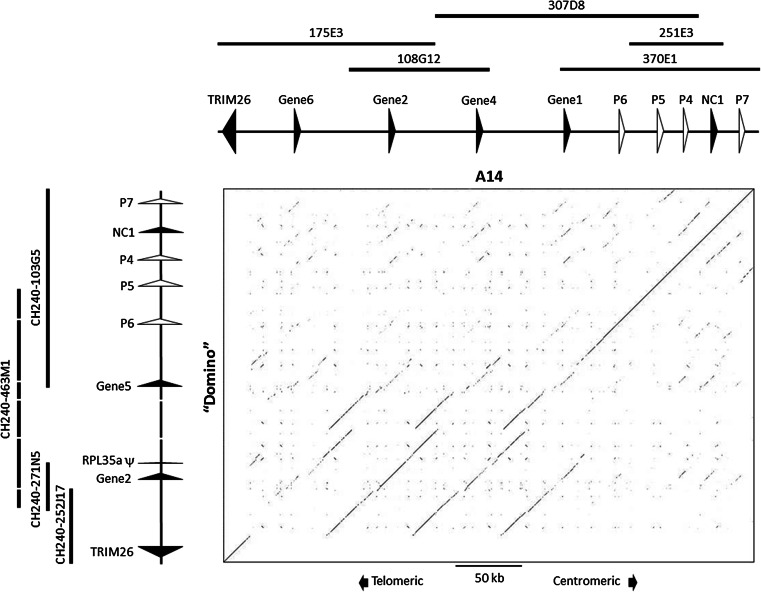
Sequence identity comparison of the assembled Hereford genome “Domino” and A14 cattle *MHC class I* regions using DOTTER (Sonnhammer and Durbin 1995) and a sliding window size of 200 bp. Putatively functional genes are shown with *closed arrows*, while pseudogenes are shown with *open arrows. Arrows* point in the direction of transcription. The positions of the BAC clones used for each assembly are indicated by each haplotype. Gaps in the CH240-463M1 assembly are indicted by *broken lines* and are not to scale. There are three additional contigs from this clone which are not shown due to their small (~1 kb) size

Detailed annotation of our A14 assembly confirmed the four previously mapped *MHC class Ia* genes upstream from *NC1* and four *MHC class I*-like pseudogenes (Fig. 2). All four *MHC class Ia* genes are separated by between 63 and 68 kb. Initially, the most upstream of these was named Gene “*Z*” and later designated 6*04001 based on phylogenetic evidence using cDNA sequence (Di Palma et al. 2002; Hammond et al. 2012). Although direct functional evidence for 6*04001 has not been published, mRNA expression has been confirmed by several studies. The A14 haplotype had previously been designated a three-gene haplotype, this data strongly suggests that the A14 haplotype possesses four functional *MHC class Ia* genes.

To compare the Hereford genome and the A14 haplotype, a dot-matrix plot of sequence identities across both haplotypes was generated using DOTTER (Sonnhammer and Durbin 1995). Although these are different haplotypes from different breeds, there was very little sequence variation between them either side of the *MHC class Ia* gene region, with *NC1* and all the pseudogenes being syntenic. The little variation observed was the processed (i.e. retrotransposed) pseudogene of *RPL35a* located downstream from *Gene 2*; however, no trace of this pseudogene was identifiable in the A14 data (Figs. 1 and 2). None of the class I pseudogenes described here showed any significant identity to *Gene 3*, the only *MHC class Ia* gene not present on either haplotype. Each *MHC class Ia* gene is located within a tandemly duplicated block of ~80 kb that each share significant sequence identity (Fig. 2). The regular, repeating nature of these similar sized blocks is suggestive of a site-specific mechanism that has facilitated cattle *MHC class I* expansion and contraction. Based on these two divergent haplotypes, this recombination appears to have occurred in a distinct region between *pseudogene 6* and *TRIM26*.

**Fig. 3:**
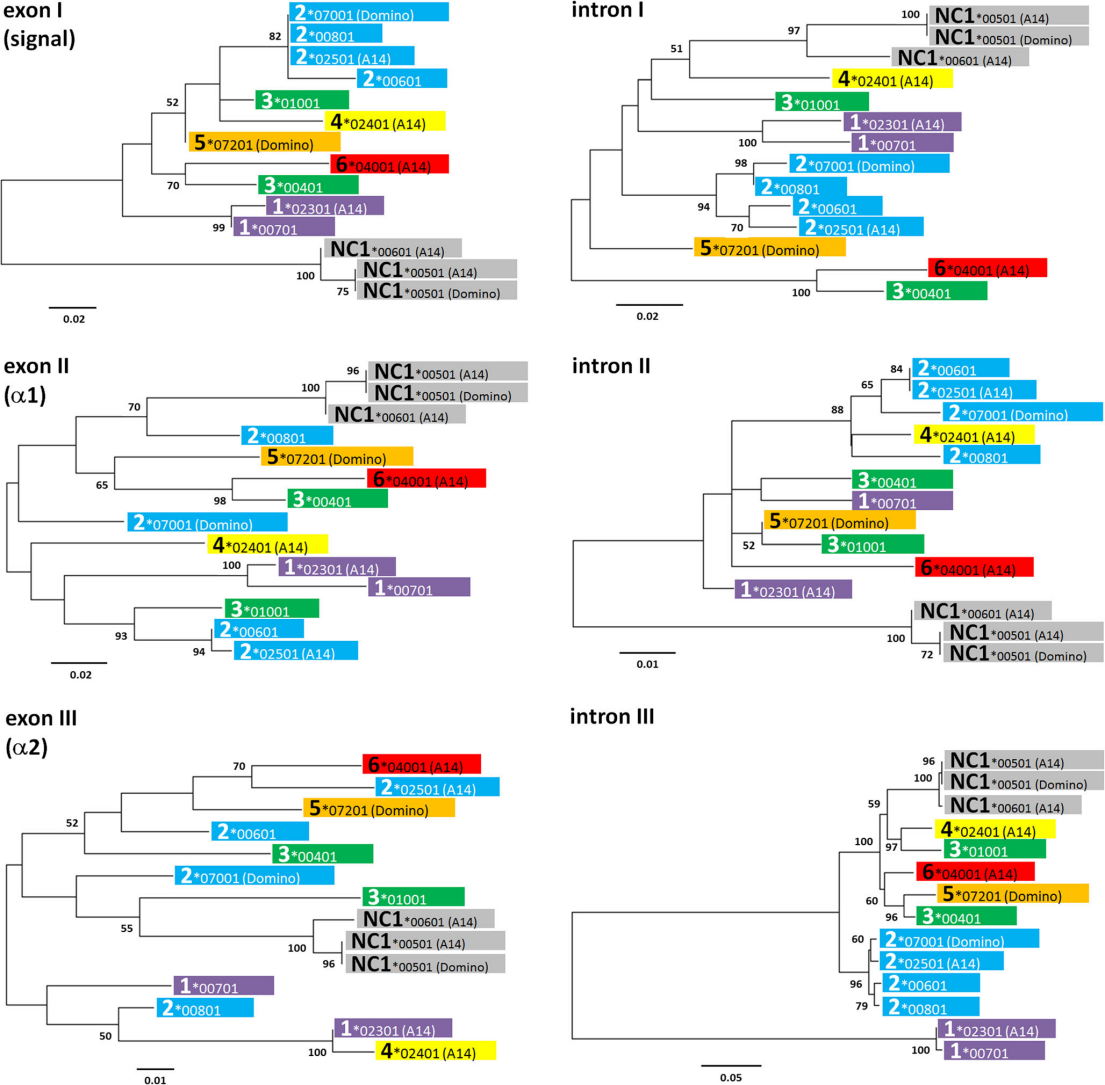
Phylogenetic relationships of cattle *MHC class I* sequences for the first three exons and introns. Genomic sequences from A14 and Hereford genome BAC clones and from GenBank (3*00401 (AF396750), 1*00701 (AF396751), 2*00601 (AF396752), 2*00801 (AF396753), and 3*01001 (AF396754)) were aligned using CLUSTAL W (Thompson et al. 1994). The tree was constructed using maximum likelihood based on the Tamura three-parameter model (Tamura 1992) within MEGA6 (Tamura et al. 2013)

**Fig. 4:**
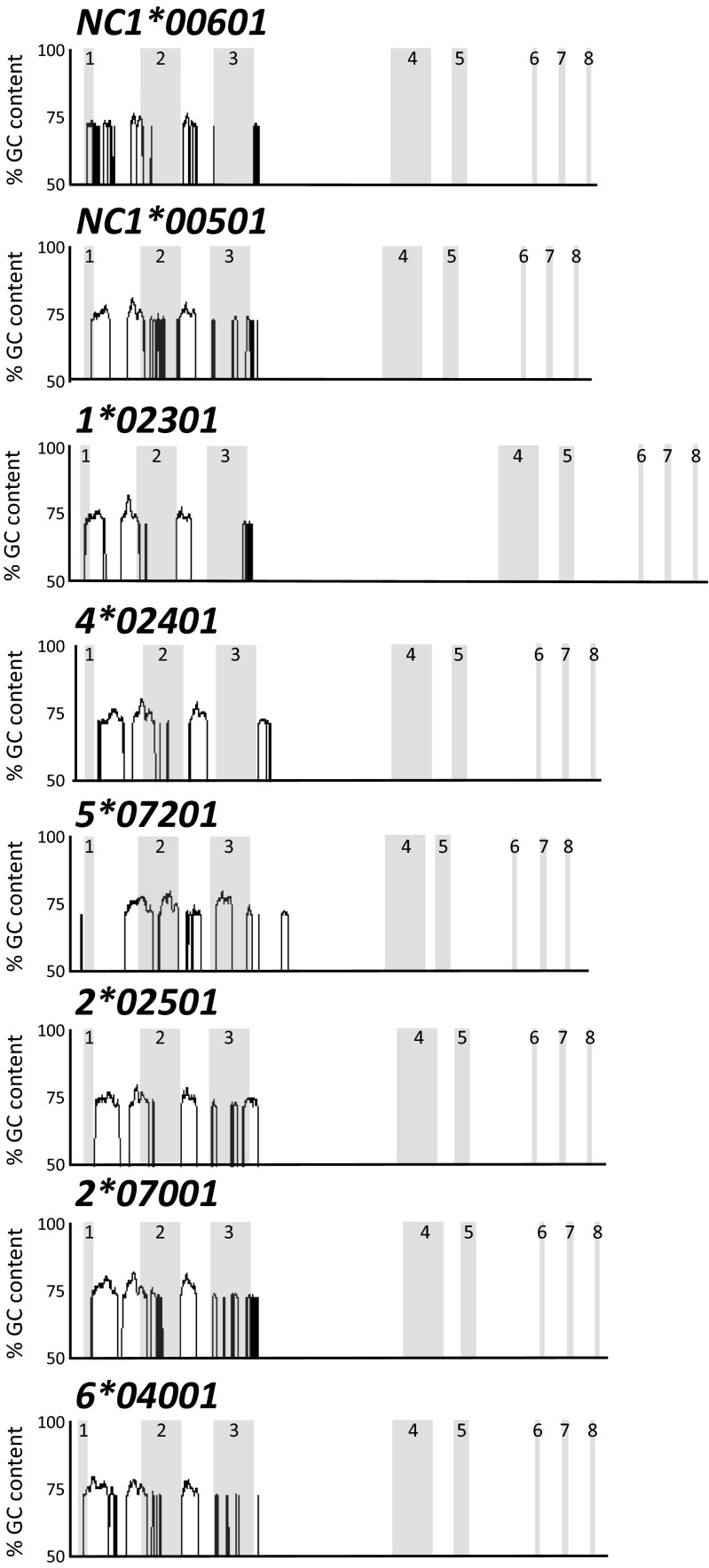
Intron/exon structure and GC content of the known complete *MHC class I* genes. Exons for each gene are *shaded* and *numbered*. Y-axis shows percent GC (from 50 to 100 %) using a sliding window of 100 bp and a 2.5-standard deviation cut-off indicating abnormally high GC content near the intron/exon boundaries of the first three exons. GC content was visualised using Artemis (Rutherford et al. 2000)

Finally, we compared the intronic and exonic sequences for all *MHC class Ia* and *class Ib* genes for which the information is available. This includes those from this manuscript, as well as five additional genes from GenBank; including two alleles of *Gene 3* (AF396750 and AF396754), two additional alleles of *Gene 2* (AF396752 and AF396753), and another allele of *Gene 1* (AF396751). Domain by domain analysis showed that sequences from the current six gene designations clade together at the more conserved 3′ end of the gene, after the α2 domain in intron 3, and confirmed the close relationship of *NC1* with the classical genes (Fig. 3). The relationship between the peptide-binding domains is far more complex, with no consistent relationships emerging with either exon or intron sequence at the 5′ end of the molecule, apart from the *NC1* sequences which always form a discrete cluster. From this limited number of sequences, it is not possible to disentangle the relative contributions of mutation and recombination to this diversification. However, it seems likely that break points in introns 1, 2, and at the 5′ of intron 3 have led to multiple gene conversion events. Interestingly, these regions all contain unusually high guanine-cytosine (GC) content (Fig. 4), possibly representing a signature of GC-biased gene conversion (Mancera et al. 2008). As well as gene conversion, diversity would be further facilitated by the haplotype block structure that the classical genes have formed.

In conclusion, this study presents the cattle genome *MHC class I* haplotype with more confidence and compares this to the first fully sequenced haplotype. We confirm the general organisation of the *MHC class I* region and have identified a haplotype structure and regions of recombination that point to the mechanisms driving diversity. These results will enable more detailed studies into the evolution and the functional consequences of this diversity across a range of haplotypes and breeds.
